# Assessing human carcinogenicity risk of agrochemicals without the rodent cancer bioassay

**DOI:** 10.3389/ftox.2024.1394361

**Published:** 2024-06-12

**Authors:** Amber Goetz, Natalia Ryan, Alaina Sauve-Ciencewicki, Caleb C. Lord, Gina M. Hilton, Douglas C. Wolf

**Affiliations:** ^1^ Syngenta Crop Protection LLC Greensboro, NC, United States; ^2^ PETA Science Consortium International e.V., Stuttgart, Germany

**Keywords:** new approach methods, weight of evidence, rodent cancer bioassay, carcinogenicity, risk assessment, agrochemical, regulatory toxicology

## Abstract

The rodent cancer bioassays are conducted for agrochemical safety assessment yet they often do not inform regulatory decision-making. As part of a collaborative effort, the Rethinking Carcinogenicity Assessment for Agrochemicals Project (ReCAAP) developed a reporting framework to guide a weight of evidence (WOE)-based carcinogenicity assessment that demonstrates how to fulfill the regulatory requirements for chronic risk estimation without the need to conduct lifetime rodent bioassays. The framework is the result of a multi-stakeholder collaboration that worked through an iterative process of writing case studies (in the form of waivers), technical peer reviews of waivers, and an incorporation of key learnings back into the framework to be tested in subsequent case study development. The example waivers used to develop the framework were written retrospectively for registered agrochemical active substances for which the necessary data and information could be obtained through risk assessment documents or data evaluation records from the US EPA. This exercise was critical to the development of a framework, but it lacked authenticity in that the stakeholders reviewing the waiver already knew the outcome of the rodent cancer bioassay(s). Syngenta expanded the evaluation of the ReCAAP reporting framework by writing waivers for three prospective case studies for new active substances where the data packages had not yet been submitted for registration. The prospective waivers followed the established framework considering ADME, potential exposure, subchronic toxicity, genotoxicity, immunosuppression, hormone perturbation, mode of action (MOA), and all relevant information available for read-across using a WOE assessment. The point of departure was estimated from the available data, excluding the cancer bioassay results, with a proposed use for the chronic dietary risk assessment. The read-across assessments compared data from reliable registered chemical analogues to strengthen the prediction of chronic toxicity and/or tumorigenic potential. The prospective case studies represent a range of scenarios, from a new molecule in a well-established chemical class with a known MOA to a molecule with a new pesticidal MOA (pMOA) and limited read-across to related molecules. This effort represents an important step in establishing criteria for a WOE-based carcinogenicity assessment without the rodent cancer bioassay(s) while ensuring a health protective chronic dietary risk assessment.

## 1 Introduction

As science evolves to capture a better understanding of a biological response, so too does the need to maintain adequate protection of human health and the environment against hazardous chemicals. A critical component of regulatory toxicology is the assessment of adverse health effects, and thus risks, in humans exposed to chemicals. Safety assessment of agrochemicals currently relies largely on animal-based toxicity testing to identify hazards and select reference values for human risk assessment. One concern in the current paradigm for the safety assessment of agrochemicals is the assessment of carcinogenicity. This is typically conducted on two separate species, rats, and mice (OECD, 2018a; 2018b), the conduct of which is driven by experience, historical precedence, and legislative requirements. The results of testing are used to set restrictions on the use, or method of use, for chemicals of concern; therefore, it is important that the choice of models, and the design of the studies, are truly protective of human health under a risk assessment approach.

Advancements in technologies and methods to assess systemic toxicity have led to an increased understanding of chemical carcinogenicity (Becker et al., 2017; Corvi et al., 2017; Dekant et al., 2017; Felter et al., 2022; Holsapple et al., 2006; OECD AOP-Wiki). It is now possible to evaluate the carcinogenic potential of a chemical using new approaches with improved human relevance (Wolf et al., 2019; Madia et al., 2021; Audebert et al., 2023). Such advances in the scientific understanding of chemically induced chronic toxicity, including carcinogenicity, provide an opportunity to modernize the evaluation of health risk from potential exposures to agrochemicals (Kavlock et al., 2018; Cohen et al., 2019; Wolf et al., 2019). Guidance exists to facilitate health-protective chemical evaluation while minimizing animal use, and only requires implementation (ECHA, 2017; Hartung, 2019; Stucki et al., 2022). Specifically, established regulatory guidance allows for scientific rationales to satisfy data requirements, promoting and optimizing the full use of existing information and focusing on the critical knowledge needed for risk assessment (US EPA 2013; APVMA, 2017; PMRA, 2021).

Characterizing carcinogenicity risk does not require development of new technologies or models, but rather leveraging the available understanding of carcinogenicity and applying existing tools in new ways (WHO, 2021; Stucki et al., 2022; Schmeisser et al., 2023). The ReCAAP Working Group, a group of experienced scientists from industry, non-governmental organizations, academia, and regulatory authorities with expertise in carcinogenicity testing, evaluation, and risk assessment, has developed a reporting framework for waiver rationales to rodent cancer bioassays for consideration in agrochemical safety assessment (Hilton et al., 2022).

The ReCAAP framework provides structure to support reporting of a WOE-based carcinogenicity assessment, including a comprehensive evaluation of all relevant data from the pesticidal mode-of-action (pMOA), physiochemical properties, metabolism, toxicokinetics, toxicological data including mechanistic data, and chemical read-across from similar registered agrochemicals. This assessment also includes an evaluation of data points related to well-known cancer MOAs such as genotoxicity, immunosuppression, and hormone perturbation. In addition, the use patterns, exposure scenario(s), and human exposure levels from the intended uses of chemicals are summarized to estimate the range of likely human exposures. The available data and known properties across structurally similar compounds (read-across analogues) are reviewed and considered for use to estimate appropriate departure points (POD) for chronic risk assessment of the active substance.

Hilton et al. (2022) performed a comprehensive evaluation of the framework by constructing WOE-based carcinogenicity assessments to support rodent cancer bioassay waiver rationales for registered agrochemicals, based on publicly available data. The availability of full data packages (including carcinogenicity studies) for these chemicals allowed for the waiver rationale to be compared back to the actual data, providing an important reference point for the framework. However, the exercise did not fully reflect the reality of the goal–to develop a waiver rationale based on the comprehensive data and information available, prior to the generation of carcinogenicity data. Agrochemical companies are in a unique position to construct a waiver rationale during the development of a regulatory data package for a new active substance, prior to knowing the results of a carcinogenicity study, and to have the waiver evaluated without influence of carcinogenicity results. Three prospective case studies are presented here, representing a range of scenarios, from a compound of a well-established chemical class with a known MOA to a compound with a limited chemical base for read-across. An overview of the WOE assessments is presented with key lessons learned.

This paper describes our efforts to evaluate the ability to use the ReCAAP WOE-based carcinogenicity assessment framework (“the framework”) to make an informed decision in developing a waiver rationale of the chronic/carcinogenicity studies in rats and mice without having the knowledge of the outcome of the bioassays. Additionally, the framework was used to estimate the POD to adequately protect human exposures from chronic risk, including cancer. The case studies will help to familiarize the reader with the benefits of implementing this modern approach to testing and evaluation.

## 2 Methodological approach

**FIGURE 1:**
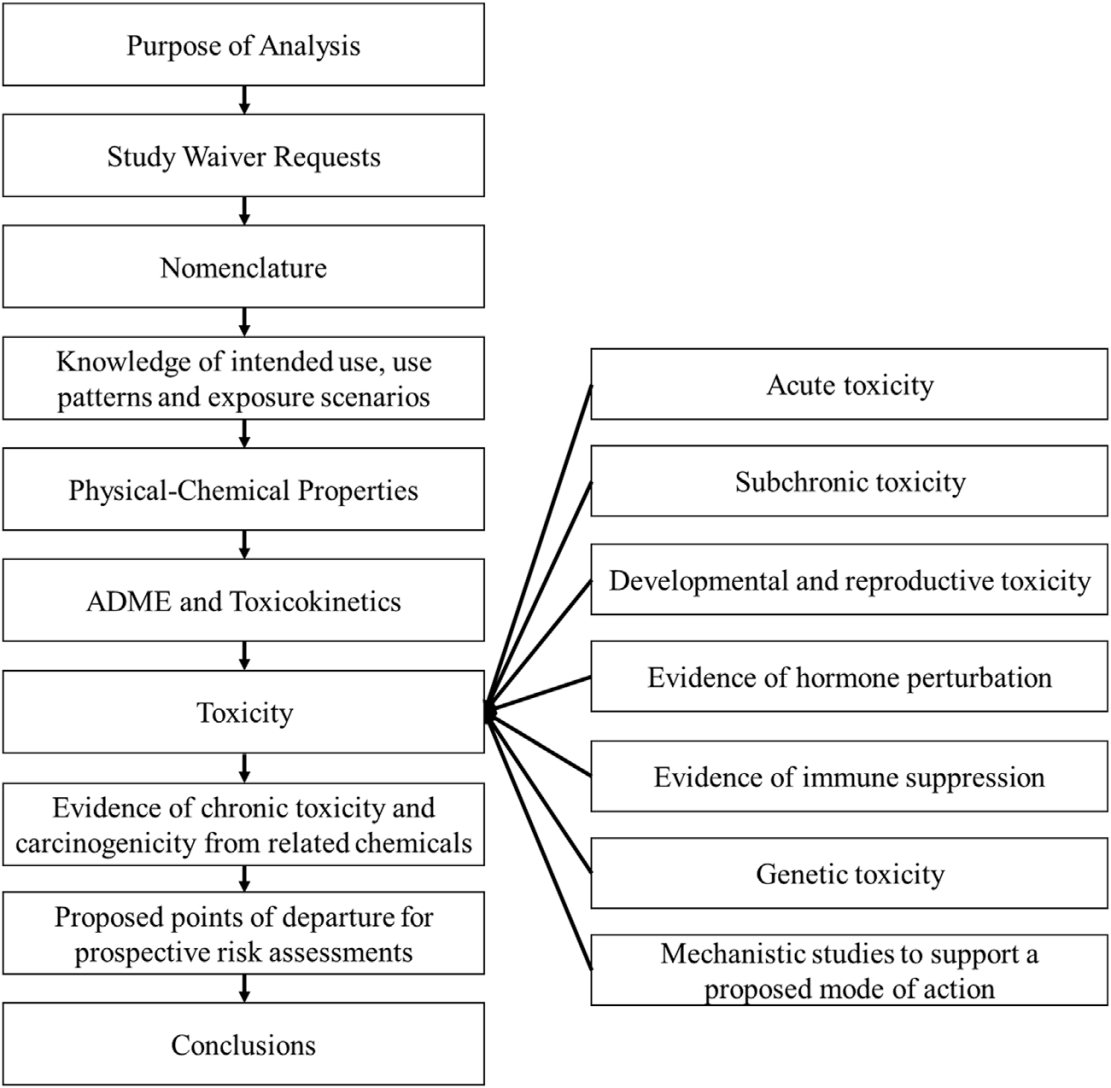
Reporting framework for the weight of evidence assessment. This workflow was used to identify and select the relevant and reliable lines of evidence used to make an informed decision in developing a waiver rationale of the chronic/carcinogenicity studies in rats and mice without having the knowledge of the outcome of the bioassays. Adapted from Hilton et al., 2022.

The overall objective with these case studies was to provide a set of prospective WOE assessments to test the robustness of this framework. The examples provided here were developed around the approach used by the United States Environmental Protection Agency (US EPA) to allow incorporation of a WOE-based approach into evaluating data for regulatory decisions (Craig et al., 2019). For each target compound, i.e., new active substance under development, the WOE assessment used the available data generated on the target compound with the exception of the chronic/carcinogenicity study. As the chronic/carcinogenicity studies were not complete at the time of the WOE assessment there was no influence on the interpretation of the WOE assessment and estimation of the POD for chronic dietary risk assessment. A read-across assessment was conducted with each case study, incorporating the relevant and reliable lines of evidence from read-across analogues, the source compounds, into the WOE assessment. Each case study applied the framework as it is laid out in the Hilton et al. (2022) paper. The outline of the workflow used to assess each individual chemical is shown in Figure 1.

### 2.1 Read-across assessment workflow

Read-across is based on the foundational principle that an association exists between structure and activity and is usually based on chemical similarity, although increasingly, also on similarities in biological effect (e.g., toxicological mode of action). Read-across seeks to inform on an endpoint outcome for an active substance (the target), where there may be a data gap, by using existing data on the same endpoint from other related substances (the sources) where a wealth of information exists (Patlewicz et al., 2013a; 2013b; 2014; 2019). In place of generating new *in vivo* toxicity testing data, read-across can be used as a line of evidence to reliably assess and address the risk, uncertainties, and deficiencies in data. The read-across assessment can be leveraged to meet regulatory data needs. The use of read-across is gaining traction as a reliable line of evidence for WOE-based safety assessments in toxicology (Low et al., 2013; Mellor et al., 2017; Alexander-White et al., 2022; Lizarraga et al., 2023). With this approach, the hazard of a target compound can be predicted from the existing toxicity data of one or many source compounds.

To identify the relevant chemical analogues for the read-across used in the case studies, structural and biological effect similarity analyses were performed. The structural similarity of these case study compounds was analyzed using an online tool ChemMine Web Tool (https://chemminetools.ucr.edu/) to examine their structural similarity to available chemistries. ChemMine is a publicly available tool available for analyzing and clustering small molecules by structural similarities, physicochemical properties or custom data types. This online tool calculates atom pair (AP) and maximum common substructure (MCS) similarities with the Tanimoto coefficient as the similarity measure, as well as identifying the largest substructure two compounds have in common. For each case study, the largest substructures in common within each class of chemistry were classified, and the AP and MCS Tanimoto scores were used to categorize the similarities. There are many software programs available for a chemical structurally-based read-across assessment, some of which are publicly available (e.g., OECD QSAR Toolbox, Morgan fingerprints, ToxPrints, US EPA’s GenRA). As such, a comprehensive read-across assessment is available for determining appropriate inclusion based on structural similarities where needed. Further assessment of bioactivity similarities between the target chemical and read-across analogues was used for the potential to refine the list of relevant read-across analogues.

In addition to the structural similarity analysis, further assessments were performed to refine the selection of relevant read-across analogues including reviews of the pesticidal mode of action (i.e., the intended target mode of action), any known toxicological MOA (i.e., off-target or unintended mode of action). There may also be subcategories within a class of chemistry with distinct differences in the off-target MOA influencing the biological response to a chemical. As described in Hilton et al., 2022, this information combined with the toxicity profile of the potential analogues was considered in refining the selection of read-across analogues and strengthened the reliability of the read-across analysis in each case study.

The toxicological data available for the read-across compounds was used to further inform prospective assessments for each case study agrochemical.

## 3 Case studies

To illustrate the use of this framework, three case studies are presented as examples of how the framework could be applied for estimating the POD for chronic and carcinogenicity risk assessment, without life-time rodent cancer bioassays. The case studies were developed to capitalize on the fact that multiple regulatory agencies can consider the incorporation of weight of evidence-based assessments in place of guideline studies for regulatory decisions (Hilton et al., 2022).

The first case study using a succinate dehydrogenase inhibitor (SDHI) fungicide provides an example with a well-understood MOA and several relevant and reliable read-across analogues. The second case study using an ACCase inhibitor insecticide provides an example with a well-understood MOA; however, the read-across chemicals were moderate in number and, although chemically similar, did not share a similar toxicity profile to the target compound. The third case study using a GABA-gated chloride channel allosteric modulator (GABA-Cl) insecticide and acaricide provides an example with a novel MOA and limited read-across analogues. Table 1 summarizes the available information for each chemical and is organized to follow the ReCAAP framework.

**TABLE 1:** Summary of case studies using the framework workflow.

Chemical/Active substance	New SDHI fungicide/Nematicide	New ACCase inhibitor insecticide	New GABA-Cl allosteric modulator Insecticide/Acaricide
Pesticidal Mode of Action	Disrupts cellular respiration through inhibition of mitochondrial enzyme succinate dehydrogenase	Disrupts fatty acid biosynthesis through inhibition of acetyl-CoA carboxylase	Disrupts inhibitory neurotransmitter signaling through allosteric modulation of GABA-gated chloride channels
Read-across chemicals	• Large number of chemicals available • 23 SDHI fungicides (FRAC Group 7) • 13 chemicals registered by US EPA • All 13 chemicals included in read-across	• Medium number of chemicals available • 23 ACCase inhibitor herbicides and insecticides • HRAC Group 1 and IRAC Group 23 • 14 chemicals registered by US EPA • 2 chemicals peer-reviewed by EFSA • 1 chemical included in a JMPR report • 3 of the 17 chemicals were included in the read-across based on structural similarity (TAs/TADs) and regulatory review by the same Agency (US EPA)	• Limited number of chemicals available • GABA-Cl antagonist insecticides (IRAC Group 2) registered by US EPA deemed structurally dissimilar and not appropriate analogues • 2 GABA-Cl allosteric modulators (IRAC Group 30) registered by US EPA; an isooxazoline and a meta-diamide • Considered 4 structurally similar related veterinary medicines • 6 chemicals included in read-across
Pharmacokinetics	Target substance is well absorbed and rapidly excreted. ADME properties were similar in both sexes and at all tested dose levels. There is no concern for bioaccumulation or toxic metabolites	Target substance is well absorbed and extensively metabolized, with no alerts for bioaccumulation or toxic metabolites. Excretion is rapid, with greater than 94% of the dose excreted within 48 h and essentially complete by 168 h. The predominant biotransformation pathway observed was via rapid and complete ester hydrolysis of the ethoxy carbonyl moiety to form the enol metabolite	Target substance is readily absorbed and extensively metabolized, with no alerts for bioaccumulation or toxic metabolites. ADME properties were similar in both sexes irrespective of dose levels, single or repeat dose, radiolabel, and sex
Relevant Assessment of Biological Effect and Response	Subchronic studies indicate liver and thyroid are target organs (increased weights and microscopic hypertrophy) with clear NOAELs established for all effects	Subchronic studies indicate decreased body weights in all tested species, thyroid effects in rats, liver effects in mice, and adverse clinical signs in dogs, with clear NOAELs established for all effects	Subchronic studies indicate several target organs in rats and mice, and no target organs in dogs. Clear NOAELs established for all effects
Evidence of hormone perturbation	No effects on reproductive performance or prenatal development. No evidence of estrogen, androgen, or steroid perturbation. Thyroid effects in rats considered secondary to liver enzyme induction. No evidence of direct thyroid perturbation	Based on the available data there is no toxicity via an endocrine MOA and thus not relevant for selection of endpoints for risk assessment. The lack of hormone measurement does not affect the WOE assessment or outcome because a hormonal MOA relevant to carcinogenicity was limited to thyroid, for which mechanistic data are available to address human non-relevance and/or justification for a margin of exposure-based approach for chronic risk assessment. Effects due to perturbations of reproductive hormones were considered adequately evaluated by the US EPA in the toxicological database, including the repeated dose, reproductive, developmental and ToxCast data	No effects on reproductive performance or prenatal development. No evidence of estrogen, androgen, or thyroid perturbation in subchronic studies. Unable to exclude steroid perturbation in the adrenal gland based on effects in rat and mouse subchronic studies, also observed for read-across chemicals
Evidence of immune suppression	No evidence of immune suppression in subchronic studies or with read-across chemicals	Chemicals used for read-across did not show signs of immunotoxicity in the T-cell dependent antibody response assays. In all available studies, there was no evidence of an immunosuppressive effect up to the highest dose level tested	No evidence of immune suppression in subchronic studies or with read-across chemicals
Genotoxicity	Not mutagenic, aneugenic, or clastogenic based on complete genotoxicity battery	Not mutagenic, aneugenic, or clastogenic based on complete genotoxicity battery	Not mutagenic, aneugenic, or clastogenic based on complete genotoxicity battery
Interpretation of Toxicity Profile	Investigative studies link the liver and thyroid effects to activation of CAR and induction of liver enzymes (including UDPGT), a well-established MOA common to most SDHI chemicals	Subchronic toxicity profile in line with ACCase inhibition. Investigative studies link thyroid effects in rats to induction of liver enzymes (including UDPGT). Adverse clinical signs in dogs identified as the most protective endpoint for risk assessment	Consistent testis effects in rat studies provide a protective endpoint for risk assessment. Varied effects observed in subchronic studies indicate clear thresholds but no clearly identified MOA.
Point of Departure	Lowest 90-day NOAEL (rat) with a 10X extrapolation factor for subchronic to chronic duration (1000X total uncertainty factor)	Lowest 90-day NOAEL (dog) with a 10X extrapolation factor for subchronic to chronic duration (1000X total uncertainty factor)	Lowest 90-day NOAEL (rat) with a 10X extrapolation factor for subchronic to chronic duration (1000X total uncertainty factor)
Chronic Risk Assessment	All use cases passed risk assessment based on margins of exposure	All use cases passed risk assessment based on margins of exposure	All use cases passed risk assessment based on margins of exposure
Conclusions on Weight of Evidence to Waive the Rodent Bioassays	High confidence that a chronic POD can be determined that is protective of all long-term effects, including cancer, without conducting the rodent bioassays	High confidence that a chronic POD can be determined that is protective of all long-term effects, including cancer, without conducting the rodent bioassays	Based on subchronic effects and tumor profiles of the read-across chemicals, weight of evidence considered not sufficient to waive the rodent bioassays

An illustration of the framework workflow. The left column lists the order of assessments utilized in the WOE, assessment. The results from each part of the analysis are briefly described for each case study.

Although the types of information, level of detail, and data interpretations will likely vary for different chemicals, these case studies are provided as examples to familiarize the reader with the format, information content, and level of detail that should be considered in a WOE assessment.

### 3.1 Succinate dehydrogenase inhibitor (SDHI)

A new agrochemical active substance has been developed which acts as an inhibitor of the mitochondrial enzyme succinate dehydrogenase; agrochemicals with a MOA involving this complex are called succinate dehydrogenase inhibitors (SDHIs). There are currently 13 SDHIs registered by the US EPA, and the data for all registered SDHIs in North America was collected for use in the read-across assessment. This target compound was selected as a case study because it has a well understood pesticidal MOA and there are several similar active substances registered, thus permitting an in-depth assessment and ability to estimate the chronic POD and cancer outcome from similar chemicals.

Short-term (28-day) and subchronic (90-day) toxicity studies in the mouse, rat and dog with this target compound primarily indicated that the liver is the target organ for toxicity. There were consistent, dose-related increases in liver weight and incidence of hepatocellular hypertrophy across the various species. Liver enzymes, including uridine diphosphate glucuronosyltransferase (UDPGT), were induced by exposure to the new SDHI. There were some noted effects on the thyroid gland (increased weight or follicular cell hypertrophy), determined to be secondary to liver effects. Additional systemic toxicity assessments demonstrated there was no evidence for genotoxicity, neurotoxicity, developmental toxicity, or reproductive toxicity for this SDHI.

The subchronic toxicity database for this new SDHI is in line with the majority of active substances in this class of chemistry. The primary target organ for SDHI inhibitors is consistently the liver across all chemicals used in the read-across assessment. The thyroid is the second most common target organ, affected by nine of the thirteen chemicals, and thyroid effects for SDHIs are considered secondary to UDPGT liver enzyme induction. The kidneys are considered a target for two of the SDHIs, and the gastrointestinal tract is considered a target for another SDHI chemical. In most cases, the rat or dog is the most sensitive species following subchronic exposure.

The read-across assessment for chronic toxicity of the presented SDHI compounds identified the same target organs observed in the subchronic toxicity studies. In general, most SDHI read-across compounds demonstrated progression of toxicity from subchronic to chronic exposure. No clear sex-specific sensitivities were identified. In the carcinogenicity assessments, treatment-related tumors were observed for nine of the thirteen compounds, as determined by the US EPA Cancer Assessment Review Committee. Consistent with the primary target organs across the class, liver and thyroid tumors were the most commonly observed. Eight SDHI compounds increased the incidence of liver and/or thyroid tumors. Two compounds increased the incidence of uterine tumors. Treatment by one compound increased incidence of brain astrocytomas, ovarian tubulostromal neoplasms, and histiocytic sarcomas of the lymphatic system. Another compound presented brain granular cell tumors in addition to thyroid tumors. For all SDHI compounds with tumors, the chronic reference dose (cRfD) was considered to provide a protective margin of exposure for carcinogenicity, with the exception of one chemical analogue that uses a q1* linear cancer risk assessment (for liver tumors). It is worth noting that a MOA framework has not been developed for this chemical, and a cancer reclassification would likely be possible if such data were generated, similar to the rest of the SDHI class. Overall, the data for SDHI chemicals indicates that liver and thyroid tumors are common and, if other tumor types do occur, a threshold-based risk assessment is considered protective of human health.

The subchronic toxicity profile of the new SDHI is consistent with the overall class of chemistry; supporting the WOE that the chronic toxicity and tumor profile will also be similar. Thus, liver, and thyroid tumors (secondary to liver enzyme induction) would be plausible for the new SDHI active substance. Considering this prediction, efforts were made to characterize the MOA, in advance of the actual observation of tumors in a carcinogenicity study. Studies demonstrated a direct activation of rat and mouse constitutive androstane nuclear receptor (CAR), increased levels of CAR-dependent gene expression, induction of liver enzymes (including UDPGT), hepatocellular hypertrophy and increased liver weight, all adding to the evidence that this new SDHI exhibits a CAR-mediated MOA (Peffer et al., 2018). It is well-established that this MOA has a clear threshold for effect, and thus does not require linear assessment of cancer risk (Meek et al., 2014). The total WOE assessment indicates that there is high certainty that a chronic POD can be determined that is protective of all long-term effects, including cancer, without conducting a chronic/carcinogenicity study.

**FIGURE 2:**
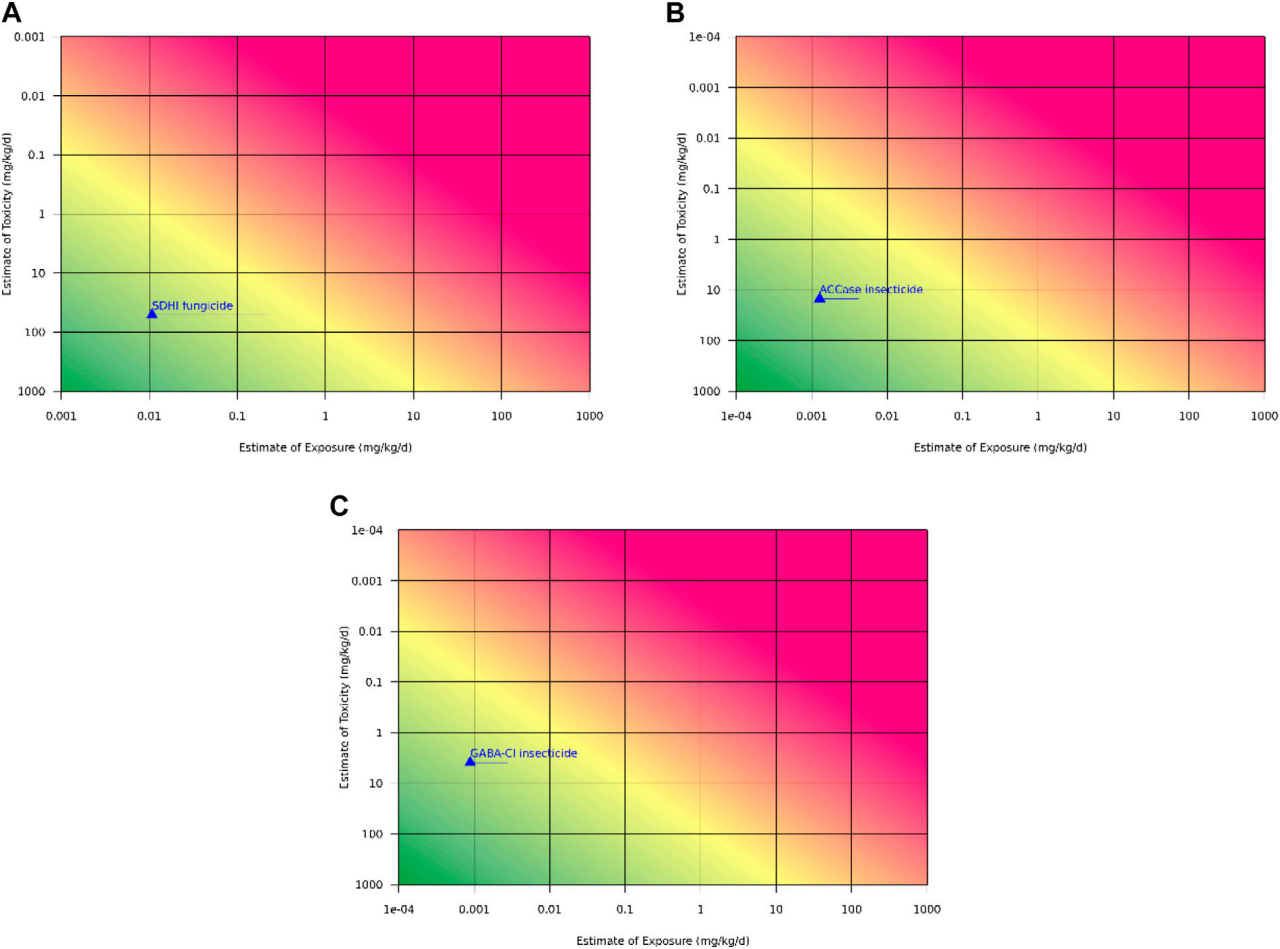
RISK21^®^ graph for predicted chronic exposure to the new active substances. The RISK21® plots evaluating the available exposure and hazard data for the safety assessment of the **(A)**. SDHI fungicide, **(B)**. ACCase inhibitor insecticide, and **(C)**. GABA-Cl insecticide. The yellow line in the RISK21® tool represents the margin of exposure between the 90-day toxicity study NOAEL (as an estimate of toxicity) and the registrant’s modeled exposure values (as estimates of exposure) generated in US EPA’s DEEM dietary risk software. The Health and Environmental Science Institute (HESI) provide RISK21® tools, which are available online through the following link: https://risk21.org/webtool/.

In the absence of a chronic study for this new SDHI chemical, it is proposed to utilize the lowest 90-day no-observed-adverse-effect-level (NOAEL) 51.1 mg/kg/day and apply an additional uncertainty factor for extrapolation from subchronic to chronic duration (Figure 2A). Based on the 90-day to chronic NOAEL ratios for the 13 SDHIs used for this comparison, a 10X uncertainty factor would be conservative. The mean of the ratios is 4.2 and the median is 3.5, indicating an extrapolation factor for study duration of 3-5X would be more appropriate to represent this class of chemistry, and still provide a chronic risk assessment that is highly protective of human health, including the risk of cancer.

Uncertainty in the SDHI case study is considered low. The mammalian toxicity and tumor profiles across the SDHI class of chemistry are strikingly similar. As liver and/or thyroid tumors would be expected for an SDHI chemical, it is conservative to assume that those tumors would result from exposure and characterize the cancer MOA proactively. The key events in the CAR/PXR pathway were investigated and assessed in line with the IPCS framework, sufficient to support evaluation of cancer risk by a regulatory agency (Boobis et al., 2006; IPCS, 2007). Further, the common CAR/PXR MOA shows a clear progression of effects with increasing duration of exposure and is known to be non-linear.

### 3.2 ACCase inhibitor insecticide (ACCase)

A new insecticide has been developed which acts as an inhibitor of acetyl-CoA carboxylase (ACCase), disrupting fatty acid biosynthesis. The ACCase class includes both herbicides and insecticides. There are currently 14 ACCase inhibitor agrochemical active substances registered by the US EPA. The availability of information for read-across, as well as the known MOA, makes this target compound a good case study.

Although ACCase is found across species, ACCase-inhibiting herbicides/insecticides do not potently inhibit mammalian, fungal, or broadleaf plant ACCase. To assess the potential use of read-across candidates, all available 23 ACCase herbicides and insecticides were initially considered. Following the structural similarity assessment, the tetronic and tetramic acid derivatives and phenylpyrazolin compounds were most structurally similar to the new ACCase inhibitor. A review of the distinguishing factors of the different ACCase chemistries and the ACCase enzyme and its physiological function was also included to ascertain any significant changes in the subcategories of this class and assess the reliability of the potential read-across analogues (Rendina et al., 1990; Délye, 2005; Yu et al., 2010; Xia et al., 2016; Takano et al., 2021).

Read-across analogues used in this case study focused on the tetramic and tetronic acid derivatives with the greatest structural similarity within the ACCase chemistry class. The ACCase herbicides were not included as they shared less structural similarity to the new insecticide, and typically insecticides show different mammalian toxicity than herbicides.

A read-across assessment for subchronic, chronic, carcinogenicity and MOA data demonstrated weak alignment across the toxicity profiles. Common effects reported in the subchronic studies were common to only two compounds, such as findings in the adrenal glands (cytoplasmic vacuolation in the cortex) in rats, mice and/or dogs following administration of spirodiclofen or spiromesifen; however, these effects were not seen with the new ACCase insecticide. Effects were observed in the male reproductive tract; including hypertrophic Leydig cells and histopathological findings in the testes, epididymis, and prostate following treatment with spirodiclofen, and decreased testis weight, testicular degeneration and vacuolation, hypospermia in the epididymis and abnormal spermatozoa following treatment with spirotetramat (EFSA, 2009; 2013); however, these effects were not seen with the new ACCase insecticide. In fact, the understanding of potential reproductive effects with this subcategory of ACCase inhibitors prompted additional evaluation during early research to allow selection of candidate compounds that did not inhibit testosterone production. Thymus atrophy was observed following treatment with spirodiclofen and spirotetramat in the dog; no effects on the thymus were seen with the new ACCase insecticide. Thyroid changes included colloid contraction and follicular cell hypertrophy following treatment with spiromesifen and the new ACCase insecticide; decreased T3 and T4 and increased TSH and thyroxine-binding capacity (TBC) following treatment with spiromesifen. Increased liver enzyme induction was also observed following treatment with spiromesifen and the new ACCase insecticide.

Short-term and subchronic exposures to this new ACCase insecticide indicated the target organs of toxicity were different for different species. The critical effects were loss of body weight in the mouse and rat, changes to the rat thyroid, increased liver weight in the mouse, and adverse clinical signs in the dog, such as body tremors and subdued behavior. The thyroid effects in the rat consisted of minimal to diffuse follicular cell hypertrophy and colloid contraction in the thyroid gland. Clear thresholds were established for all critical effects, and the adverse clinical observations in dogs were considered protective of other effects observed in the subchronic studies. Additional systemic toxicity assessment demonstrated there was no evidence for genotoxicity, neurotoxicity, developmental toxicity, or reproductive toxicity for this ACCase insecticide.

The subchronic toxicity profile for this target compound is in line with results indicative of effects on lipid biosynthesis which produced systemic effects such as decreased body weight, decreased cholesterol and triglycerides, adverse clinical signs, and changes to the liver. The systemic effects and NOAEL values from the subchronic studies identified the dog as the most appropriate species for estimating the POD for risk assessments if dosed over a longer time interval.

The initial strategy to include all ACCase chemistries in the read-across assessment was a conservative approach based on the improvements made to this class of chemistry over time. Despite these improvements, the read-across assessment includes all available data on the relevant analogues to provide a thorough assessment.

Due to the liver and thyroid effects observed in the short-term and subchronic toxicity studies in the rat with the new active substance, it was investigated whether the effects were secondary to liver enzyme induction, to better understand if liver and thyroid tumors would be plausible for the new ACCase insecticide. Considering this prediction, efforts were made to proactively characterize the MOA, in advance of the actual observation of any tumors in a carcinogenicity study. Mechanistic research excluded direct effects on thyroid peroxidase (TPO) inhibition and demonstrated a dose concordance between the rat thyroid effects and induction of liver enzymes (including UDPGT activity), hepatocellular hypertrophy, and increased liver weights, providing a weight of evidence that any liver and thyroid tumor potential of this new ACCase insecticide would be secondary to liver enzyme induction. It is well-established that this MOA has a clear threshold for effect, and thus does not require linear assessment of cancer risk. A chronic POD can be determined that is protective of all long-term effects, including cancer.

In the absence of a chronic study for this new ACCase insecticide, it was proposed to utilize the lowest 90-day NOAEL 15 mg/kg/day and apply an additional uncertainty factor for extrapolation from subchronic to chronic duration (Figure 2B). Based on the 90-day to chronic NOAEL ratios for the 3 ACCase chemicals used for this comparison, a 10X uncertainty factor would be conservative. The mean of the NOAEL ratios is 5.2 and the median is 5.6, thus, an uncertainty factor of 5-6X would be more appropriate to represent this class of chemistry and still provide a chronic risk assessment that is highly protective of human health, including the risk of cancer.

Uncertainty in the ACCase case study is considered low. The mammalian toxicity profile was in line with the results indicative of effects on lipid biosynthesis which were observed in the read-across analogues. Although three structurally similar read-across compounds were identified within the pesticidal MOA ACCase inhibitors, there was no common MOA except for the UDPGT induction MOA for one analogue and the target compound. This was based on an evaluation of the publicly available toxicological datasets for all ACCase compounds, which demonstrated slightly different target organs between the chemical classes. The read-across compounds were used in this case to decrease the uncertainty of predicting chronic toxicity and carcinogenicity with the target compound. This increases confidence in a safety assessment, as effects can be observed and characterized in sub-chronic studies, without the need to progress to studies of longer duration. Defining a threshold for precursor effects in sub-chronic studies would be protective of any tumor formation or chronic toxicity, and thus form the basis for a health-protective risk assessment.

### 3.3 GABA-gated chloride channel allosteric modulator (GABA-Cl)

A novel agrochemical active substance has been developed which acts as a broad-spectrum insecticide and acaricide. This compound is classified as a gamma-aminobutyric acid-gated chloride channel allosteric modulator (GABA-Cl; IRAC Group 30) which acts at a site different from known conventional GABA-Cl antagonists such as fiproles and cyclodienes (IRAC Group 2; Blythe et al., 2022; Dayan, 2019). The group of chemicals from IRAC Group 2 was determined to be structurally dissimilar and not appropriate for use in the read-across evaluation. There were two other GABA-Cl allosteric modulators registered by the US EPA, fluxametamide and broflanilide. One is an isoxazoline similar to the new active substance under development and the other is a meta-diamide; both were included in the read-across evaluation. The isoxazoline chemistry has also been used in the veterinary drug industry, and therefore four analogues were selected from that chemical space. This new GABA-Cl modulator was selected as a case study because it has a novel MOA, with limited read-across analogues, and therefore estimating the chronic POD or cancer outcome based on similar chemicals was more challenging.

In the veterinary drug industry (i.e., non-food uses), carcinogenicity studies are not warranted when there is no concern for genotoxicity (EMA 2013, 2015, 2017a, 2017b). In the case of the six analogues selected for read across, all were demonstrated to be non-mutagenic and non-clastogenic. There were no structural alerts for genotoxicity, and there were no proliferative or pre-neoplastic changes in the subchronic rat toxicity studies. -Therefore, chronic toxicity and carcinogenicity data were available only for the two agrochemicals (fluxametamide and broflanilide), thus the read-across assessment analysis for long-term effects focused on the results from these two compounds. For both compounds, the rat was the most sensitive species and there was not a clear sex difference. For the dietary studies with fluxametamide, there was a common effect noted in the gastrointestinal tract which included gross pathology (increased incidence of abnormally pale color duodenum and jejunum) and an increased incidence of enterocyte epithelial vacuolation of the jejunum. Increased incidences of thyroid follicular cell adenoma in male rats and hepatocellular adenoma in male mice were observed in the carcinogenicity studies, albeit at doses approaching the limit dose; a genotoxic MOA was excluded as unlikely, and a threshold dose in the risk assessment was considered appropriate (Food Safety Commission, 2020). The US EPA determined that a non-linear approach using the chronic reference dose would account for all toxicities, including carcinogenicity (US EPA, 2020a; 2020b). Based on the overall toxicology profile for broflanilide, the target organs were the adrenal glands (rats, mice, dogs) and ovaries (rats and mice). No effects were observed in the mouse carcinogenicity study. In rats, there were testicular Leydig cell adenomas, ovarian luteomas and granulosa cell tumors, uterine adenocarcinomas, and adrenal cortex carcinomas observed in the carcinogenicity study (US EPA, 2020b).

Short-term and subchronic exposures to the new GABA-Cl modulator indicated the rat was the most sensitive species, with clear NOAELs established for all effects in all species. There was no clear consistent target organ of toxicity, as the critical effects varied across species. The target organs identified in the rat were the adrenal glands, duodenum, kidneys, liver, testes, and epididymides, while the target organs in the mouse were the adrenal glands, duodenum, liver, spleen, and thymus. There were no target organs identified in the dog. There were clear and protective thresholds for all effects, based on dose levels with no observed effects. Additional toxicity assessment demonstrated there was no evidence for genotoxicity, neurotoxicity, developmental toxicity, and no evidence of potential carcinogenicity based on data points related to well-known cancer MOAs such as genotoxicity and immunosuppression. An effect on the hypothalamus-pituitary axis could not be completely ruled out due to effects in the adrenals. The most sensitive effect across the toxicity database was testicular tubular degeneration in rats, evident in the one-generation and two-generation reproductive studies, and the 90-day subchronic study; however, there was no effect on reproductive function for males. Consistent NOAELs for the testicular effects were observed across all studies, with no evidence of progression or lower effect levels with longer duration exposure, allowing for an estimation of a POD suitable for use in chronic risk assessment. Overall, the toxicity profile indicated this new GABA-Cl modulator would be unlikely to generate treatment-related tumors in rats or mice if a long-term set of studies were conducted. Using the lowest 90-day NOAEL 3.9 mg/kg/day as the POD and applying an additional factor of 10X for extrapolation from subchronic to chronic duration, the chronic reference value was well above the anticipated human exposures, indicating a health-protective chronic risk assessment would be possible without rodent cancer bioassays (Figure 2C).

Following the technical peer reviews, it was noted that the relevance of the histopathological findings in liver, renal cortical tubular epithelia, and adrenal zona fasciculata vacuolation could have been discussed in greater depth to better support a waiver rationale. For example, an important finding for the GABA-Cl case study was the fact that the adrenal changes were limited to zona fasciculate vacuolation and adrenal gland hypertrophy. No adrenal proliferative lesions were observed despite the presence of vacuolation. This was important as the histopathological distinction between adrenal hyperplasia and adrenal neoplastic changes in the rat is not necessarily easy to differentiate. The same feedback applied to the adrenal findings in mice. To strengthen this case study, additional data that provided more detailed understanding of the effects in the adrenal gland were recommended by the ReCAAP collaborative reviewers of the case study.

Concerning toxicokinetics, this new GABA-Cl modulator and its metabolites did not appear to bioaccumulate. In summary, the target organs for subchronic exposure included the adrenal glands (rat and mouse), liver (rat and mouse), kidneys (rat), testes (rat), duodenum (rat and mouse), spleen (mouse) and thymus (mouse). Carcinogenicity studies were only available for two of the related chemicals, and multiple tumor types were observed in the carcinogenicity studies for both chemicals. In addition, there were indications in the read-across for potential disruption of the hypothalamic-pituitary adrenal (HPA) axis, based on hypertrophy of adrenal zona fasciculata seen in mice and rats in the toxicity databases. Based on the number of organs affected in the subchronic studies and the inconsistent tumor profiles of the read-across chemicals, there was limited confidence that a waiver rationale would be acceptable for risk management, and presently, would not be sufficient to fulfill the regulatory data requirements. This case study highlights the need for additional steps to develop mechanistic cell-based assays and computational models that can acceptably address such uncertainties.

### 3.4 Comparison to chronic/carcinogenicity study results

As already noted, the opportunity to develop these prospective case studies for three new agrochemicals was unique, because the exercise was blinded to the results of the guideline chronic/carcinogenicity studies that were eventually conducted in both mice and rats to fulfill current data requirements for registration. For all three new agrochemicals described above, the rodent bioassays did not show any evidence of carcinogenicity not predicted by the framework assessment. For the SDHI chemical, mouse liver tumors were observed (although at a low incidence considered marginally treatment-related). As the CAR-mediated MOA had been demonstrated through mechanistic studies, a threshold-based endpoint for chronic toxicity was considered protective of all long-term effects in humans, including cancer. No treatment-related tumors were observed in either rats or mice for the ACCase inhibitor or the GABA-Cl modulator. The application of the ReCAAP framework to these chemicals resulted in waiver rationales and estimated chronic PODs that were equally or more conservative than the actual results of the rodent bioassays, illustrating that human health-protective chronic and carcinogenicity risk assessments do not necessarily require long-term animal data.

## 4 Key learnings

The intended value of these Syngenta case studies was to provide an opportunity for reviewers from the ReCAAP Work Group to test the application of the ReCAAP framework for three new agrochemicals, without any knowledge of the rodent bioassay outcomes. The aggregated reviewer feedback from this exercise underlined the core strengths of the framework to support a WOE-based assessment of chronic and carcinogenicity risk without the rodent bioassays. Through this exercise, several key learnings were identified, including the advantage of using read-across and mode-of-action information to support the WOE, as well as the need for transparency in the selection and justification of information used in the WOE. In the following, we summarize some of the key learnings and recommendations from this exercise, to support confidence in using the ReCAAP framework for future application.

### 4.1 Read-across

One of the lessons learned with these case studies included the benefits of using a consistent approach in read-across to available guideline studies and research models and strengthening the reliability of comparing findings in known toxicological profiles. A thorough discussion of the information considered in the read-across approach was important to support the selection of chemical analogues. Read-across assessments for these case studies were conducted by evaluating structural similarity, which is a common approach for analogue selection for industrial and cosmetic chemicals. Agrochemicals, unlike industrial chemicals, typically have a well-characterized toxicity profile, as well as known on-target mode of action (i.e., pesticidal mode of action). For agrochemicals, similarity of biological effects (off-target mode of action), in addition to similarity of known on-target pesticidal mode of action, is considered an important consideration in selecting analogues for read-across evaluation. Biological similarity among chemicals is a scientifically acceptable concept, but its application requires robust justification (Escher et al., 2019; Rovida et al., 2020). Given the breadth of information supporting the read-across, it is useful to provide data tables that show a normalized magnitude of change (e.g., percent change relative to control) for similar critical effects, to aid interpretation of biological significance across toxicity studies and databases for a new active substance and read-across chemicals.

One of the challenges that arose was data availability for structurally similar chemicals. Without publicly-available data, it may not be possible to include all relevant chemicals in the read-across exercise. Likewise, reliable regulatory reviews may not be available for all chemicals, or reviews may be available from different agencies with differing interpretations. In the case of differing regulatory conclusions, choices must be made as to which positions to use in the read-across, and these decision points should be transparent and documented in the read-across assessment. While there may have been a larger library of structurally similar chemicals available for each of the case studies, only a subset was available in the public domain and in regulatory reviews. One weakness of limiting the chemical analogues (source chemicals) in this way is the potential to introduce bias for compounds with lower toxicity (i.e., chemicals that have successfully achieved development and registration). It was also recommended to apply a structured evaluation approach, a globally harmonized approach for consistency in assessing the relevance and reliability of a read-across analogue (Boobis et al., 2006; Moustakas et al., 2022).

It was noted in the reviewers’ feedback that mechanistic understanding of the treatment-related effects of an active substance and structurally similar chemicals, and the ability to compare the toxicity profiles in terms of dose-response and duration, provided the strongest read-across evidence to estimate a protective POD for human health risk assessment.

### 4.2 Mode of action

Another key strength of the framework was the emphasis on using mechanistic understanding of carcinogenicity (such as mode of action, adverse outcome pathways, and human relevance) to evaluate human risk, including targeted investigative studies if necessary. Specifically, mode of action research supports a better understanding of the biological response; through such understanding, the human relevance, and health-protective thresholds (e.g., occurrence of key events) can be identified.

To bring increased consistency to MOA evaluation it was recommended during the technical peer reviews that possible MOAs and/or AOPs be considered systematically during the WOE assessment. and included in the framework to prompt the registrants to include this in the assessment. To develop a sustainable framework, relevant MOAs and/or AOPs to evaluate would be helpful to streamline the WOE and should be adaptable to evolve over time. It is important to consider the relevant MOA that may drive the chronic toxicity risk of a chemical; however, this framework is not designed to be prescriptive on which tools or studies must be used. As highlighted in this ReCAAP framework, each weight of evidence assessment should be evaluated on a case-by-case basis, using scientifically sound relevant MOA based on the available data. Additional feedback on the MOA research for each case study highlighted the strength of the available data. Some potential MOAs were accepted as adequately addressed (e.g., rat thyroid effects secondary to liver enzyme induction) while certain alternative MOAs were considered only partially addressed (e.g., receptor-mediated MOA) or not sufficiently addressed (e.g., altered apoptosis). Further research may be required to strengthen the MOA assessment of certain effects of concern, and iterative engagement with the Regulatory Agencies could help to identify database uncertainties and ensure an acceptable MOA assessment strategy.

### 4.3 Transparency

Another key learning from the case studies was the need for transparency in the rationale used to assess the safety of the target compound with the available data and read-across analogues. Depending on the individual case study, various lines of evidence may be deemed more or less informative and relevant to the overall WOE. For instance, read-across may be highly useful in some cases (e.g., in the SDHI case study), but in other cases may not be strong enough to predict chronic and carcinogenicity risk for certain endpoints (e.g., in the GABA-Cl case study). While there may be a common pesticidal MOA across the chemicals used in the read-across, the off-target effects and biological response may be different. Thus, the selection of read-across analogs must be adequately justified.

The MOA data is generally expected to be an impactful line of evidence. The use of GLP OECD guideline studies (i.e., regulatory approved study design and quality), as well as any publicly available relevant information on the chemical analogues, strengthened these assessments.

The Syngenta prospective case study reviews provided useful feedback and guidance on options to improve and increase the acceptance of the ReCAAP framework. Recommendations from the ReCAAP Work Group technical reviews included the provision of a consistent and structured approach in the methodology used for read-across, including well-articulated criteria and a transparent decision tree used for selecting read-across chemicals. Additionally, presenting more information on similarity grouping, mechanistic data, and mammalian mode of action research to support the read-across rationale would increase the confidence and strengthen the ability to compare toxicity profiles, and thus inform on an endpoint outcome for a new target active substance.

## 5 Next steps for this framework

The WOE-based ReCAAP framework is designed to integrate several different types of toxicological evidence, which can include regulatory-required guideline toxicity studies, chemical read-across, and mechanistic new approach methods (e.g., *in vitro* assays, toxicogenomics). In some cases, the selection of and confidence in each line of evidence will have addressed the outcomes of concern in all data streams. In others, there may only be data available. In each case, the data integration process considers the findings described in the qualitative and/or quantitative data selection and the certainty of the evidence for each outcome, to determine conclusions that directly address the human health and safety of the target compound. Looking forward, as the guidance for this approach is further developed, it may be useful to involve a matrix-based approach (e.g., a matrix describing how the confidence in the lines of evidence are combined to reach different hazard conclusions, or techniques for eliciting expert knowledge) to support the needs for regulatory risk assessment. In general, higher confidence in the lines of evidence results in stronger conclusions. The use of mechanistic data is particularly valuable to support evaluation of biological plausibility with hazard conclusions or extrapolation approaches in dose-response assessments.

The three Syngenta prospective case studies presented here demonstrate the utility of the developed ReCAAP framework to a) assess confidence in evaluating potential for carcinogenicity without the conduct of the rodent cancer bioassays, b) estimate a POD for chronic risk assessment, and c) assess the relevance and reliability of the lines of evidence identified and selected for the read-across and WOE analysis. This modern approach can be applied to a range of different endpoints that are of common concern for safety assessment. Moreover, the framework is demonstrated to be transparent and scientifically sound, such that it is ready to implement into human health risk assessment. Further, with the key learnings during the WOE assessment, feedback and learnings from the technical reviews, and recommendations presented herein, this approach can be refined further to address all uncertainties and facilitate the development of guidance for more efficient, fit-for-purpose, human-relevant and equally health-protective safety, and risk assessment of chemicals. Efforts are now underway to establish a decision-making framework in the form of an Integrated Approach to Testing and Assessment (IATA) for guiding data collection, evaluating reliable and relevant information, and the decision-making process. As registrants and regulators continue to gain experience with the application of this framework to new chemicals, similar to our experience through these case studies, we anticipate further progress and acceptance of WOE rationales to support regulatory decision-making and protection of human health, without requiring long-term animal testing to evaluate chronic and carcinogenicity risk.

## Abbreviations

ACCase, Acetyl-CoA carboxylase inhibitor; ADME, Absorption, Distribution, Metabolism and Excretion; AOP, Adverse Outcome Pathway; CAR, Constitutive Androstane Receptor; cRfD, chronic reference dose; GABA-Cl, GABA-gated chloride channel allosteric modulator; LOAEL, Lowest Observed Adverse Effect Level; MOA, biological Mode of Action^1^; pMOA, pesticidal Mode of Action; NOAEL, No Observed Adverse Effect Level; POD, Point of Departure; PXR, Pregnane X receptor; ReCAAP, Rethinking Carcinogenicity Assessment for Agrochemicals Project; SDHI, Succinyl dehydrogenase inhibitor; T3, Triiodothyronine; T4, Thyroxine; TBC, Thyroxine-binding capacity; TPO, Thyroid Peroxidase inhibition; TSH, Thyroid stimulating hormone; UDPGT, Uridine diphospho-glucuronyltransferase; WOE, Weight of evidence.
